# Cerebellar Transcranial Direct Current Stimulation in Spinocerebellar Ataxia Type 3: a Randomized, Double-Blind, Sham-Controlled Trial

**DOI:** 10.1007/s13311-022-01231-w

**Published:** 2022-05-02

**Authors:** Roderick P. P. W. M. Maas, Steven Teerenstra, Ivan Toni, Thomas Klockgether, Dennis J. L. G. Schutter, Bart P. C. van de Warrenburg

**Affiliations:** 1 Department of Neurology, Donders Institute for Brain, Cognition, and Behaviour, Radboud University Medical Center, Nijmegen, the Netherlands; 2 Department for Health Evidence, Biostatistics Section, Radboud University Medical Center, Nijmegen, the Netherlands; 3 Donders Institute for Brain, Cognition, and Behaviour, Radboud University, Nijmegen, the Netherlands; 4 Department of Neurology, University of Bonn, Bonn, Germany; 5 German Center for Neurodegenerative Diseases (DZNE), Bonn, Germany; 6 Experimental Psychology, Helmholtz Institute, Utrecht University, Utrecht, the Netherlands

**Keywords:** Spinocerebellar ataxia type 3, Transcranial direct current stimulation, Randomized controlled trial, Cerebellar brain inhibition, Transcranial magnetic stimulation, Scale for the Assessment and Rating of Ataxia

## Abstract

Repeated sessions of cerebellar anodal transcranial direct current stimulation (tDCS) have been suggested to modulate cerebellar-motor cortex (M1) connectivity and decrease ataxia severity. However, therapeutic trials involving etiologically homogeneous groups of ataxia patients are lacking. The objective of this study was to investigate if a two-week regimen of daily cerebellar tDCS sessions diminishes ataxia and non-motor symptom severity and alters cerebellar-M1 connectivity in individuals with spinocerebellar ataxia type 3 (SCA3). We conducted a randomized, double-blind, sham-controlled trial in which twenty mildly to moderately affected SCA3 patients received ten sessions of real or sham cerebellar tDCS (i.e., five days per week for two consecutive weeks). Effects were evaluated after two weeks, three months, six months, and twelve months. Change in Scale for the Assessment and Rating of Ataxia (SARA) score after two weeks was defined as the primary endpoint. Static posturography, SCA Functional Index tests, various patient-reported outcome measures, the cerebellar cognitive affective syndrome scale, and paired-pulse transcranial magnetic stimulation to examine cerebellar brain inhibition (CBI) served as secondary endpoints. Absolute change in SARA score did not differ between both trial arms at any of the time points. We observed significant short-term improvements in several motor, cognitive, and patient-reported outcomes after the last stimulation session in both groups but no treatment effects in favor of real tDCS. Nonetheless, some of the patients in the intervention arm showed a sustained reduction in SARA score lasting six or even twelve months, indicating interindividual variability in treatment response. CBI, which reflects the functional integrity of the cerebellothalamocortical tract, remained unchanged after ten tDCS sessions. Albeit exploratory, there was some indication for between-group differences in SARA speech score after six and twelve months and in the number of extracerebellar signs after three and six months. Taken together, our study does not provide evidence that a two-week treatment with daily cerebellar tDCS sessions reduces ataxia severity or restores cerebellar-M1 connectivity in early-to-middle-stage SCA3 patients at the group level. In order to potentially increase therapeutic efficacy, further research is warranted to identify individual predictors of symptomatic improvement.

**Supplementary Information:**

The online version contains supplementary material available at 10.1007/s13311-022-01231-w.

## Introduction

Spinocerebellar ataxia type 3 (SCA3), also called Machado-Joseph disease, is the most common cause of dominantly inherited ataxia worldwide [1]. It results from a trinucleotide repeat expansion in the *ATXN3* gene and is characterized by gradually progressive incoordination of gait, limbs, speech, and eye movements that usually manifests alongside a variety of extracerebellar signs. Impairments in daily activities due to motor deterioration and cognitive decline are not only paralleled by a reduced quality of life and high prevalence rates of depression and fatigue but also pose a significant economic burden on healthcare services and society in general [2–7]. Although considerable progress has been made in elucidating the clinical and radiological evolution of SCAs, we still lack disease-modifying therapies that target the underlying genetic defects [8–11]. Likewise, the number of evidence-based symptomatic treatment options for ataxia currently remains rather limited, emphasizing the need to explore alternative therapeutic strategies [12].

Cerebellar transcranial direct current stimulation (tDCS) has recently emerged as a non-invasive neuromodulation technique capable of influencing perceptual, motor, cognitive, and emotional behavior [13–15]. Empirical evidence suggests that its effects are exerted through modification of Purkinje cell excitability [16]. Considering that abnormal firing patterns of Purkinje cells are among the earliest pathophysiological changes of many cerebellar disorders, including SCA3, administration of weak electric currents to target the cerebellar cortex may be a promising approach to mitigate ataxia severity and cognitive deficits [17]. Interestingly, repeated sessions of tDCS could potentially prolong the positive after-effects of a single session to days, weeks, or even months by generating cumulative changes in synaptic efficacy and enhancing functional connectivity of the cerebellum with the primary motor cortex (M1) and cortical association areas [14, 15, 18].

Three randomized controlled trials from the same group have recently demonstrated substantial clinical improvement in ataxia patients with mixed etiologies following a two-week regimen of daily cerebellar anodal or cerebello-spinal tDCS sessions [19–21]. In the first two studies, the reported reduction in ataxia severity was found to persist throughout the three-month follow-up period, accompanied by a restoration of functional cerebellothalamocortical tract connectivity [20, 21]. In their third study, the investigators extended the randomized, double-blind phase with an open-label phase, demonstrating the add-on effect of a second round of cerebello-spinal tDCS after three months on both motor and cognitive outcomes [19]. Albeit encouraging, further trials are required to evaluate the effects of similar treatment protocols in etiologically homogeneous cohorts. In this study, we aimed to investigate if repeated sessions of cerebellar anodal tDCS induce (1) sustained improvements in ataxia and non-motor symptom severity and (2) alterations of cerebellar-M1 connectivity in patients with SCA3.

## Methods

### Study Design and Participants

The SCA3-tDCS study was an investigator-initiated, randomized, double-blind, sham-controlled, parallel-group trial that took place at the Radboud University Medical Center. A paper describing the detailed study protocol has previously been published [22]. Patients received five daily sessions of real or sham cerebellar tDCS per week for two consecutive weeks. Outcomes were evaluated directly after the last session (T1) and after three (T2), six (T3), and twelve months (T4) of follow-up by the same examiner according to a fixed schedule for each time point. In this way, factors that might have negatively affected performance, such as fatigue, were kept similar between both groups. We also examined the short-term results of single-session tDCS.

Eligible participants were adults with a confirmed pathogenic repeat expansion in the *ATXN3* gene and mild to moderate ataxia, defined as a Scale for the Assessment and Rating of Ataxia (SARA) score between 3 and 20 at a recent (pre-study) visit [23]. We deliberately aimed to study the effects of tDCS as add-on intervention reflecting current daily practice and therefore did not exclude individuals who received physical therapy or took medication. One patient in the intervention group used a calcium channel blocker (amlodipine) because of hypertension, which might have influenced the efficacy of tDCS [24]. Patients were excluded if they had epilepsy, a history of brain surgery, metallic implants in or near the skull, a pacemaker, significant comorbidities that interfered with daily activities, (suspicion of) pregnancy, or a skin condition that affected the location of electrode placement.

The trial protocol has been approved by the local ethics committee (CMO region Arnhem-Nijmegen). Written informed consent was obtained from every participant according to the Declaration of Helsinki. The study was registered in the Netherlands Trial Register (NL7321) on October 8, 2018.

### Randomization and Blinding

SCA3 patients who fulfilled the eligibility criteria were allocated in a 1:1 ratio to receive real or sham tDCS using the randomization module of the data management system Castor EDC. Permuted block randomization stratified by ataxia severity was used with random computer-generated block sizes of either two or four. The procedure was conducted by an independent physician who subsequently selected a five-digit code from the neuroConn tDCS user manual that corresponded with the mode of stimulation to which that participant was assigned. A specific code was never picked more than once. In this way, patients, investigators, and outcome assessors were masked to treatment allocation.

### Transcranial Direct Current Stimulation

Administration of cerebellar tDCS occurred by means of two 7 × 5 cm rubber electrodes that were connected to a neuroConn constant current stimulator. Electrode montage was consistent with the set-up applied by Benussi et al. [21, 25]. The horizontally oriented anode was encased in a saline-soaked sponge and centered over the midline 2 cm below the inion, while the cathode was placed over the right deltoid muscle. Impedance levels were kept below 5 kΩ throughout the stimulation. Elastic gauzes and tape were used to secure electrode positions.

The procedure for real and sham tDCS was identical. Every stimulation session started with a ramp-up period of 30 s in which the current intensity was gradually increased. During real stimulation, a constant current of 2 mA was subsequently delivered for 20 min, followed by a fade-out time of 30 s. By default, the sham condition contained 40 s of real stimulation, which was followed by a similar fade-out time and 1160 s of continuous impedance control without any stimulation. Participants’ thoughts about group assignment and possible side effects were explored at T1.

### Clinical Outcome Measures

Ataxia severity was assessed using the SARA [23]. Individual items were videotaped and rated by two experienced investigators who were masked to randomization status and point in time. In case of discrepancies, consensus was reached by discussion. Absolute change in SARA score at T1 was chosen as the primary outcome measure. We additionally determined changes in axial, appendicular, and speech items, as well as the proportion of patients per group with clinically meaningful improvement, which was defined by a decrease in SARA score of at least 1.5 points [26]. The meaningfulness of changes in SARA score from a patient perspective was explored at T1 using the Patient Global Impression of Change scale. Lastly, participants were asked if and how long they had noticed general effects of cerebellar tDCS at home between T1 and T2.

The 8 m walk test (8MWT), nine-hole peg test (9HPT), and PATA repetition task were used as secondary motor endpoints to evaluate gait speed, manual dexterity, and articulation speed, respectively. Average scores per outcome measure were calculated from two trials. The extent of extracerebellar involvement was quantified by the Inventory of Non-Ataxia Signs (INAS) [27].

Changes in activities of daily living (ADL), health-related quality of life, depressive symptoms, mood states (i.e., fatigue, vigor, tension, anger, and depression), physical activity, and direct medical costs were ascertained by part II of the Friedreich Ataxia Rating Scale (FARS), EQ-5D-5L, Patient Health Questionnaire-9 (PHQ-9), 32-item Dutch version of the Profile of Mood States (POMS), International Physical Activity Questionnaire (IPAQ, parts 1 and 4), and iMTA Medical Consumption Questionnaire (iMCQ), respectively [28–33].

Effects of cerebellar tDCS on cognitive deficits were assessed by total score and number of failed items at the cerebellar cognitive affective/Schmahmann syndrome scale (CCAS-S). In order to attenuate practice effects in follow-up administrations, different versions of this instrument were applied [34].

### Static Posturography

Besides descriptive, semi-quantitative SARA stance ratings, static posturography was used at baseline and T1 to derive more objective markers of balance. Participants stood barefoot on a 60 × 40 cm force platform (Motekforce Link) equipped with four piezoelectric sensors at its corners that quantified ground reaction forces and excursions of the resultant center of pressure (CoP) in the anteroposterior (AP) and mediolateral (ML) directions with a sampling frequency of 1000 Hz. Intermalleolar distance was individually adjusted in increments of 5 cm such that the narrowest stance width — and therefore most challenging standing position — was obtained for each patient. Fixed lines of adhesive tape were applied to the force platform to facilitate these measurements and ensure nearly identical foot placement at baseline and T1. Toes were always pointing forward. Participants performed three trials of 30 s duration in which they were instructed to stand as quietly as possible, fix their gaze upon a target straight ahead, and hold their arms relaxed along their body. Breaks between trials were allowed as long as necessary but in practice lasted less than 2 min at a time.

To eliminate the effects of slightly unequal foot positions between measurements, an established normalization procedure was conducted using MATLAB (R2018a, Mathworks) in which mean values of CoP position and speed in both directions were calculated per trial and taken as zero point [35, 36]. Raw data were passed through a second-order Butterworth bandpass filter (cut-off frequencies 0.15 Hz and 6 Hz) to remove disturbing low-frequency baseline drift and high-frequency signal components [37]. Root mean square values of the time series were computed as markers of average sway amplitude and velocity in AP and ML directions. Total CoP path length complemented the list of force plate endpoints. Similar to the SARA stance item, static posturography measures were determined in the best out of three trials. Considering the possibility of intertrial variability, we also calculated mean values of these parameters from all three attempts.

### Cerebellar Brain Inhibition

The functional integrity of the cerebellothalamocortical tract at baseline and after two weeks of tDCS was ascertained by a paired-pulse transcranial magnetic stimulation (TMS) protocol called cerebellar brain inhibition (CBI). In healthy individuals, a significant decrease in motor evoked potential (MEP) amplitude is observed when a test stimulus (TS) over M1 is preceded by a conditioning stimulus (CS) over the contralateral cerebellar hemisphere within an interval of 5 to 7 ms [38]. Patients with a compromised cerebellothalamocortical pathway exhibit less or no CBI [39, 40].

The methodology of CBI assessment has been described in detail elsewhere [22, 41]. In brief, we used a 110 mm double-cone coil and 70 mm figure-of-eight coil, both connected to a Magstim BiStim^2^ module, to deliver conditioning stimuli over the right cerebellar hemisphere and test stimuli over the hand area of the left M1, respectively. In line with most previous reports, the double-cone coil was positioned 1 cm below and 3 cm lateral to the inion on the line joining the external auditory meatus and oriented in such a manner to induce an upward secondary electric current in the brain [42]. The handle of the figure-of-eight coil pointed backwards and laterally in a 45° angle with respect to the midsagittal plane. Patients wore a tight rubber cap to allow marking of the “hotspots” for both coils and ensure their consistent placement throughout the experiment. MEPs were recorded from the first dorsal interosseus muscle of the right hand through a pair of Kendall H69P electrodes positioned in a belly-tendon montage.

The resting motor threshold (rMT) was defined as the minimum stimulus intensity required to elicit MEPs (≥ 50 μV or visible by contraction) in five out of ten trials during complete muscle relaxation. Intensity of cerebellar conditioning stimuli was set at 90% rMT obtained from M1, while intensity of test stimuli was adjusted to evoke MEPs with a peak-to-peak amplitude between 0.5 and 1.0 mV [20, 21, 43–46].

Ten TS-only trials, ten CS-TS trials with a 5 ms interstimulus interval (ISI), ten CS-TS trials with a 3 ms ISI, and ten CS-TS trials with a 10 ms ISI were presented in a pseudorandomized sequence. Mean amplitudes of MEPs were assessed per ISI and expressed as a ratio of unconditioned MEP amplitudes in TS-only trials.

### Statistical Analysis

Assuming a predetermined sample size of twenty participants who each have five measurements and a partial *η*^2^ value of 0.46 [21], which corresponds to a large effect size *f* of 0.92, this study would have an estimated power of 0.99 to detect significant differences when SARA score is used as the primary endpoint (G*Power 3.1).

Initially, we planned to perform repeated measures ANOVA to evaluate the effects of tDCS on clinical scores [22]. However, missing data from some participants for some outcomes at the final, one-year follow-up visit due to the COVID-19 pandemic would have led to complete loss of these subjects from the analysis. We therefore employed linear mixed models that include these patients and take into account the correlations between repeated observations. This decision was made prior to unblinding. Both subject-specific models with random intercepts and slopes that model the trajectories of individual patients and marginal models that model the means at each visit with an unstructured variance–covariance matrix for the residuals using generalized least squares regression were considered. Fixed effects were included for time point, intervention, and, in case of the primary endpoint, also baseline SARA and IPAQ scores. Outcomes at each visit, including baseline, and change from baseline scores were considered as the dependent variable. The best-performing model (i.e., subject-specific versus marginal and absolute score versus change from baseline) was selected for every single endpoint by a visual assessment of goodness-of-fit and comparison of Akaike’s information criterion values (see the Supplementary data for a more detailed description of the various models).

For outcome measures that were determined at two time points, we used independent samples *t*-tests or Mann–Whitney *U* tests to compare delta scores. Between-group differences in proportions were evaluated by Fisher’s exact tests. Statistical analyses were conducted in SPSS (version 25). The significance level was set at *p* < 0.05 (two-sided).

## Results

### Study Participants

Between October 29, 2018 and April 1, 2019, twenty SCA3 patients were enrolled and randomly assigned to real or sham tDCS (Supplementary Fig. 1). No relevant differences were identified at baseline between both treatment groups in any of the clinical and demographic variables (Table 1). There was no loss to follow-up, but unfortunately in-clinic visits of the last four participants at T4 (March/April 2020) could not take place due to the COVID-19 pandemic. Nonetheless, SARA scores were successfully obtained through home video recording by extensively instructed family members [47], and questionnaires were completed electronically, leaving missing data only for INAS count, 8MWT, 9HPT, and the PATA repetition task.

**Table 1:** Baseline clinical and demographic characteristics of SCA3 patients in the real tDCS and sham tDCS group

	**Sham tDCS (*n* = 10)**	**Real tDCS (*n* = 10)**
**General characteristics**		
Age (y)	51.4 ± 9.8	52.4 ± 10.8
Age of onset (y)	42.6 ± 8.8	45.2 ± 9.9
Disease duration (y)	8.8 ± 6.2	7.2 ± 4.7
CAG repeat length, expanded allele	67.3 ± 3.1	67.8 ± 3.8
Sex (% male)	5 (50)	7 (70)
**Motor outcomes**		
SARA score	12.5 ± 4.7	11.3 ± 3.2
Gait	2.6 ± 0.8	2.4 ± 0.5
Stance	2.0 ± 1.4	1.6 ± 0.8
Sitting	1.0 ± 0.5	0.9 ± 0.3
Speech	1.7 ± 0.7	2.2 ± 0.6
Finger chase	1.3 ± 0.4	0.9 ± 0.3
Nose-finger test	0.9 ± 0.7	0.9 ± 0.5
Fast alternating hand movements	1.1 ± 0.9	0.9 ± 0.9
Heel-shin slide	2.0 ± 0.7	1.6 ± 0.9
8 m walk test (s)	6.8 ± 2.8	5.7 ± 0.9
Nine-hole peg test, dominant hand (s)	31.1 ± 9.5	32.1 ± 7.4
Nine-hole peg test, non-dominant hand (s)	33.9 ± 7.8	33.3 ± 5.0
PATA repetition rate	28.1 ± 6.1	27.2 ± 5.9
INAS count	5.8 ± 1.5	5.9 ± 1.7
**Neuropsychological measures**		
CCAS-S, total score	83.4 ± 11.2	80.3 ± 7.0
CCAS-S, number of failed tests	2.9 ± 2.3	3.2 ± 1.2
**Patient-reported outcome measures**		
EQ-5D VAS score	67.6 ± 19.0	77.8 ± 12.4
EQ-5D utility index	0.76 ± 0.21	0.80 ± 0.11
PHQ-9 score	3.6 ± 2.5	3.8 ± 3.4
POMS fatigue	5.2 ± 4.9	4.5 ± 3.9
POMS depression	1.9 ± 2.6	3.3 ± 2.7
POMS anger	1.6 ± 3.5	3.6 ± 4.2
POMS tension	1.7 ± 1.3	1.8 ± 2.0
POMS vigor	11.2 ± 4.0	12.4 ± 4.4
FARS ADL score	11.9 ± 3.5	12.6 ± 4.1
Medical consumption, direct costs (euro)	718.7 ± 513.5	636.0 ± 408.6
Physical activity (MET minutes per week)	2361 ± 1851	2742 ± 2512
**Neurophysiological outcome measures**		
TMS – rMT (%)	40.6 ± 4.0	40.2 ± 3.6
TMS – CBI	0.87 ± 0.17	0.94 ± 0.07

*CAG* cytosine-adenine-guanine; *SARA* Scale for the Assessment and Rating of Ataxia; *INAS* Inventory of Non-Ataxia Signs; *PHQ-9* Patient Health Questionnaire-9; *POMS* Profile of Mood States; *MET* Metabolic Equivalent of Task; *FARS* Friedreich Ataxia Rating Scale; *ADL* activities of daily living; *CCAS-S* cerebellar cognitive affective syndrome scale; *rMT* resting motor threshold; *CBI* cerebellar brain inhibition

In general, cerebellar tDCS was safe and well-tolerated. Mild headache, transient tiredness, a burning feeling underneath the electrodes, and dizziness for several seconds after stimulation were all reported once by patients who received sham tDCS. Mild headache and an itching sensation below the electrodes were mentioned by one and two participants in the intervention group, respectively. Furthermore, we observed two focal skin lesions at the site of stimulation in a patient with a tattoo on his right shoulder, as described in more detail elsewhere [48]. Four participants experienced a transient feeling of pressure underneath the double-cone coil during TMS.

Mean group values for each outcome measure at T0 to T4 are presented in Supplementary Tables 1 and 2. Between-group differences per time point, including 95% confidence intervals (CIs), are summarized in Table 2. Baseline SARA and IPAQ scores were eventually removed as covariates in the analysis of the primary endpoint because a likelihood-ratio test showed that this did not yield a statistically significantly worse fit.

**Table 2:** Estimated treatment effects (i.e., differences between patients in the intervention group and patients in the sham group) and 95% confidence intervals for each outcome measure at the various time points

	** T1** **2 weeks**	** T2** **3 months**	** T3** **6 months**	** T4** **12 months**
**Ataxia severity**				
SARA score^a^	0.15 [− 1.2; 1.5]	0.05 [− 1.8; 1.9]	− 0.50 [− 2.4; 1.4]	− 0.60 [− 2.2; 1.0]
SARA score, percent change^a^	0.11 [− 11.4; 11.6]	4.57 [− 15.1; 24.2]	− 0.66 [− 20.1; 18.8]	− 1.51 [− 19.2; 16.2]
SARA axial^b^	− 0.10 [− 1.1; 0.9]	− 0.20 [− 1.3; 0.9]	− 0.20 [− 1.4; 1.0]	− 0.40 [− 1.4; 0.6]
SARA appendicular^a^	0.45 [− 0.3; 1.2]	0.45 [− 0.7; 1.6]	0.30 [− 0.9; 1.5]	0.20 [− 1.0; 1.4]
SARA speech^b^	− 0.30 [− 0.7; 0.1]	− 0.30 [− 0.9; 0.3]	− 0.70 [− 1.3; − 0.1]^*^	− 0.50 [− 1.0; 0.03]
**Quantitative motor tests and extracerebellar signs**				
8MWT^a^ (s)	0.42 [− 0.4; 1.3]	0.16 [− 0.6; 1.0]	0.02 [− 0.6; 0.6]	− 0.7 [− 1.6; 0.2]
9HPT, dominant hand^b^ (s)	1.12 [− 1.9; 4.1]	− 0.81 [− 4.6; 3.0]	− 0.71 [− 4.3; 2.9]	− 0.14 [− 5.1; 4.9]
9HPT, non-dominant hand^a^ (s)	0.15 [− 2.6; 2.9]	− 0.24 [− 2.8; 2.4]	− 1.89 [− 5.3; 1.5]	− 0.19 [− 4.4; 4.1]
PATA rate^a^	1.40 [− 1.6; 4.4]	4.40 [− 0.02; 8.8]	2.05 [− 1.7; 5.8]	3.19 [− 0.3; 6.7]
INAS count^b^	− 0.68 [− 1.5; 0.1]	− 1.68 [− 3.0; − 0.4]^*^	− 2.58 [− 3.7; − 1.5]^*^	− 0.98 [− 2.3; 0.4]
**Neuropsychological outcome measures**				
CCAS-S, total score^a^	0.70 [− 5.2; 6.6]	− 0.30 [− 6.5; 5.9]	− 0.30 [− 6.2;5.6]	− 0.30 [− 6.8; 6.2]
CCAS-S, failed tests^b^	0.50 [− 0.9; 1.9]	− 0.40 [− 1.5; 0.7]	− 0.20 [− 1.3; 0.9]	0.0 [− 1.4; 1.4]
**Patient-reported outcome measures**				
EQ-5D VAS score^a^	− 5.60 [− 12.2; 1.0]	− 8.90 [− 18.8; 1.0]	− 11.50 [− 24.9; 1.9]	− 6.30 [− 15.7; 3.1]
EQ-5D utility index^c^	− 0.046 [− 0.1; 0.06]	− 0.055 [− 0.2; 0.06]	− 0.059 [− 0.2; 0.09]	− 0.033 [− 0.2; 0.1]
PHQ-9 score^a^	− 0.80 [− 3.1; 1.5]	− 0.30 [− 2.3; 1.7]	0.60 [− 1.4; 2.6]	0.016 [− 1.9; 1.9]
POMS fatigue^a^	− 0.20 [− 3.3; 2.9]	0.80 [− 1.7; 3.3]	− 1.20 [− 3.6; 1.2]	− 0.15 [− 2.0; 1.7]
POMS depression^b^	− 0.20 [− 1.4; 1.0]	0.10 [− 1.6; 1.8]	− 0.10 [− 2.9; 2.7]	0.53 [− 0.9; 2.0]
POMS anger^b^	− 0.40 [− 2.7; 1.9]	0.50 [− 1.5; 2.5]	0.80 [− 2.1; 3.7]	0.53 [− 2.5; 3.6]
POMS tension^b^	− 0.30 [− 1.7; 1.1]	0.60 [− 1.6; 2.8]	− 0.10 [− 1.4; 1.2]	− 0.39 [− 2.0; 1.2]
POMS vigor^a^	0.0 [− 3.7; 3.7]	− 1.20 [− 3.7; 1.3]	− 1.70 [− 3.9; 0.5]	− 1.53 [− 4.2; 1.2]
FARS ADL score^a^	− 0.50 [− 2.1; 1.1]	− 1.20 [− 3.0; 0.6]	− 0.80 [− 2.5; 0.9]	− 1.00 [− 3.0; 1.0]
iMCQ score (euro)	-	-	-	45 [− 357; 448]
IPAQ^b^ (MET minutes per week)	-	− 599 [− 1730; 532]	-	− 57 [− 1236; 1122]
**Neurophysiological outcome measures**				
TMS – rMT (%)	0.80 [− 0.9; 2.5]	-	-	-
TMS – CBI	− 0.10 [− 0.3; 0.1]	-	-	-

A detailed explanation of the statistical models is provided in the Supplementary data

*SARA* Scale for the Assessment and Rating of Ataxia; *8MWT* 8 m walk test; *9HPT* nine-hole peg test; *INAS* Inventory of Non-Ataxia Signs; *CCAS-S* cerebellar cognitive affective syndrome scale; *PHQ-9* Patient Health Questionnaire-9; *POMS* Profile of Mood States; *FARS* Friedreich Ataxia Rating Scale; *ADL* activities of daily living; *iMCQ* Institute for Medical Technology Assessment (iMTA) Medical Consumption Questionnaire; *IPAQ* International Physical Activity Questionnaire; *MET* Metabolic Equivalent of Task; *TMS* transcranial magnetic stimulation; *rMT* resting motor threshold; *CBI* cerebellar brain inhibition

^*^Significant difference between both groups (*p* < 0.05)

^a^Change from baseline model with no adjustment for other variables

^b^Change from baseline model, adjusting for the baseline value of an outcome measure

^c^Model including baseline and four post-baseline measurements

### Motor Outcomes

**Fig. 1:**
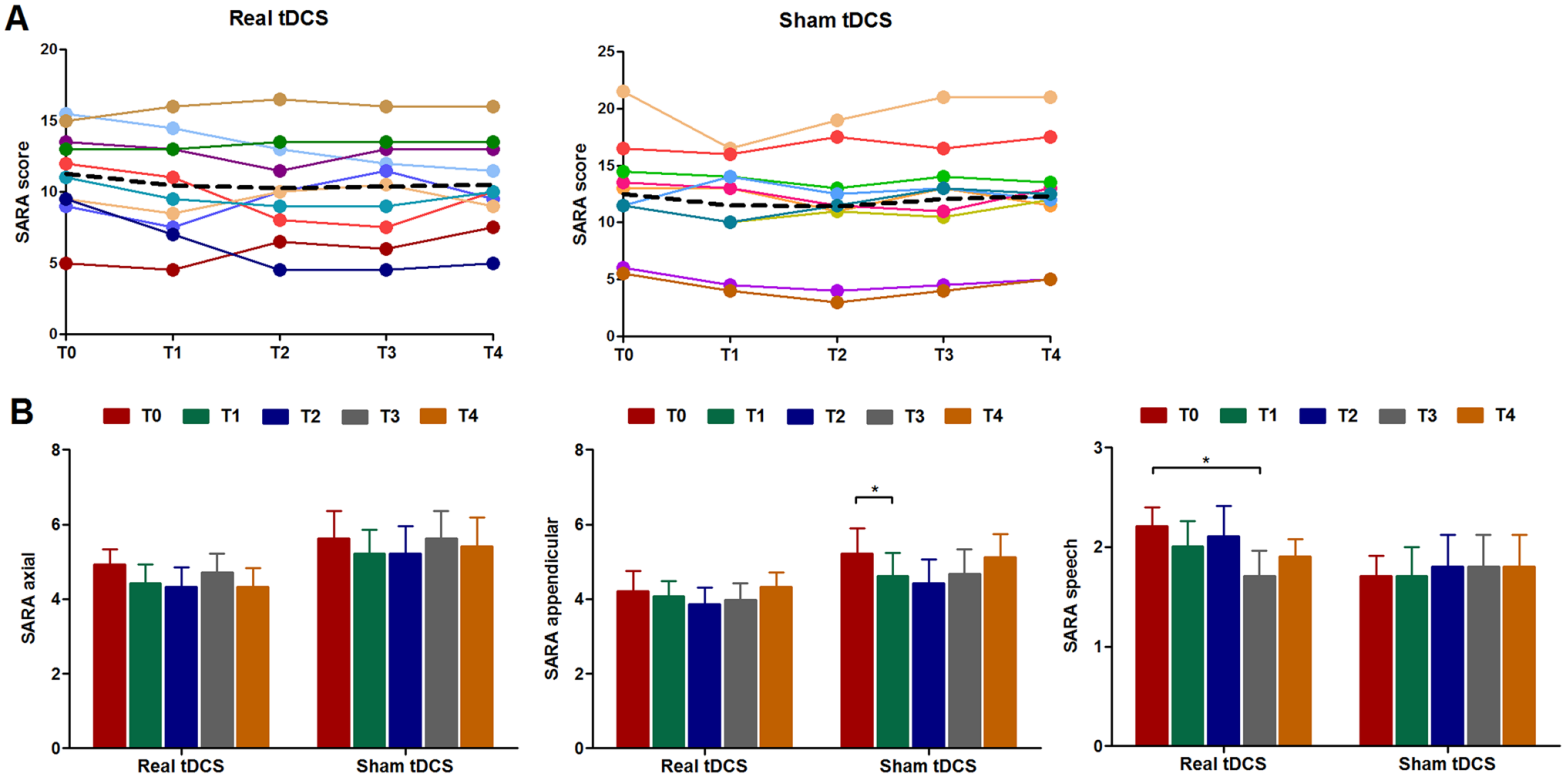
Scale for the Assessment and Rating of Ataxia (SARA) scores at different time points. Panel (**A**) shows trajectories of individual SCA3 patients in the real and sham tDCS group at baseline (T0) and after two weeks (T1), three months (T2), six months (T3), and twelve months (T4). The dashed black lines represent mean group scores. There were no significant differences in delta SARA score between both groups at any of the time points. Panel (**B**) shows SARA axial, appendicular, and speech domain scores. For SARA speech, a treatment effect of real tDCS was observed at T3, with between-group differences close to significance at T4. There were no treatment effects for axial and appendicular subscores. ^*^Significant change from baseline (*p* < 0.05)

Absolute change in SARA score from baseline did not differ between both treatment arms at T1, T2, T3, and T4 (all *p* values > 0.40). Individual SARA trajectories of participants throughout the study are illustrated in Fig. 1A. Compared to the baseline assessment, mean SARA score decreased by 0.85 points (95% CI: − 1.8 to 0.1) at T1 in the real tDCS group and by 1.0 point (95% CI: − 2.0 to − 0.02) in the sham tDCS group (Supplementary Table 3). Clinically relevant improvement, as defined by a reduction of at least 1.5 points, was found in 30% of patients who had received real tDCS and in 50% of those who had received sham tDCS (*p* = 0.65). Raising this threshold from 1.5 to 2, 3, or 4 points yielded no relevant changes. After one year of follow-up, 30% of participants in the intervention group still scored ≥ 1.5 points less than at baseline (two of them even 4 and 4.5 points less) versus 10% of participants in the sham group (*p* = 0.58). Closer inspection of SARA’s functional domains over time revealed a treatment effect of real tDCS at T3 (95% CI of difference from baseline between both groups: − 1.32 to − 0.07, *p* = 0.03) and a trend at T4 (95% CI: − 1.03 to 0.03, *p* = 0.065) for speech, but not for axial and appendicular subscores (Fig. 1B).

**Fig. 2:**
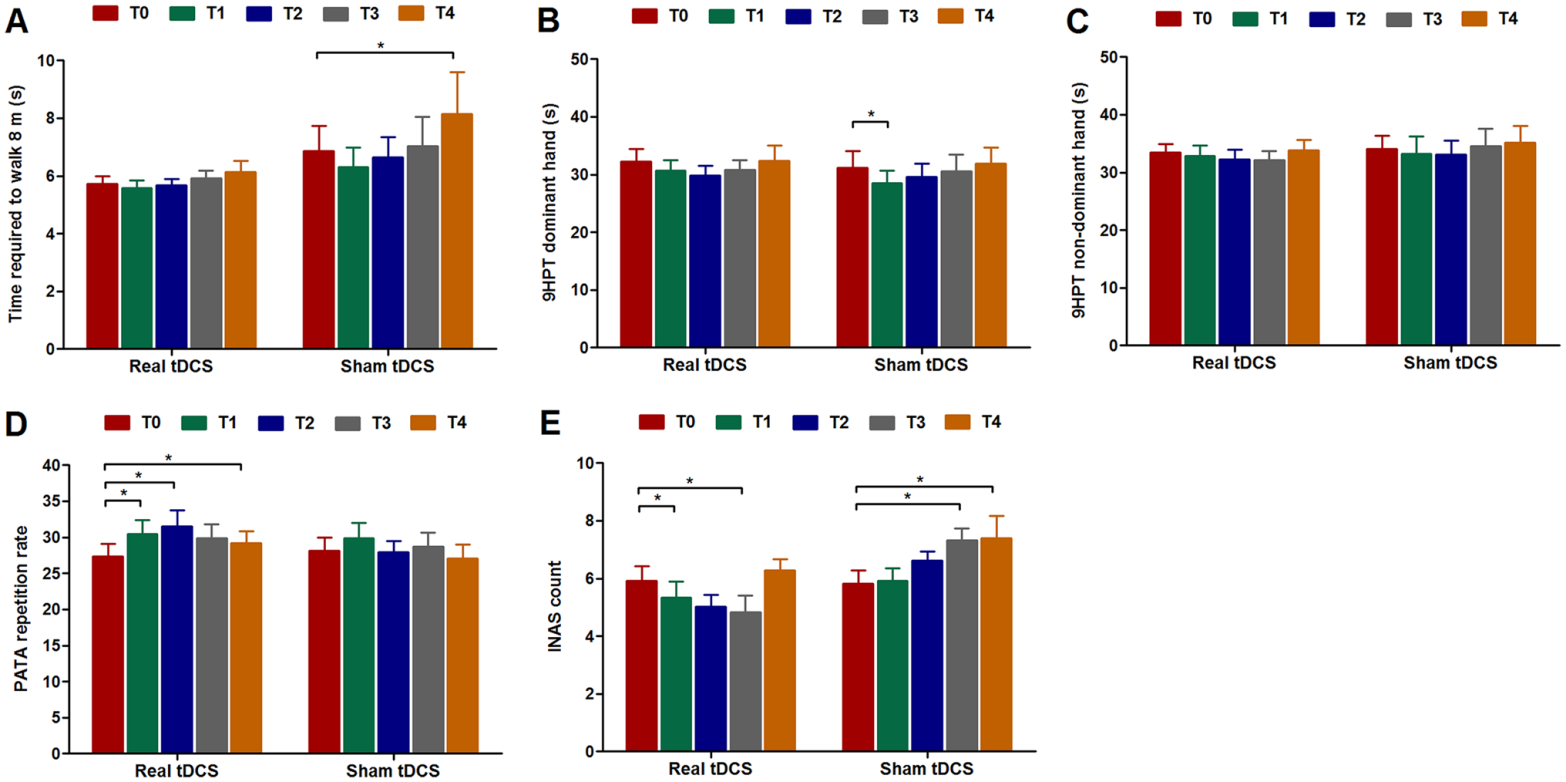
Secondary motor outcome measures at different time points. Shown are group mean scores for the 8 m walk test (**A**), 9-hole peg test (9HPT) performed with the dominant hand (**B**), 9HPT performed with the non-dominant hand (**C**), PATA repetition task (**D**), and Inventory of Non-Ataxia Signs (INAS) count (**E**) at baseline (T0) and after two weeks (T1), three months (T2), six months (T3), and twelve months (T4). A treatment effect of real tDCS was observed for INAS count at T2 and T3. Between-group differences in delta PATA repetition rate were close to significance at T2 and T4. There were no treatment effects for the 8 m walk test or 9-hole peg test. ^*^Significant change from baseline (*p* < 0.05)

Regarding extracerebellar signs, we found between-group differences in absolute change in INAS count from baseline at T2 (95% CI: − 2.99 to − 0.38, *p* = 0.016) and T3 (95% CI: − 3.66 to − 1.51, *p* < 0.001) in favor of real tDCS (Fig. 2E). Improvements mainly involved the dystonia (40%), urinary dysfunction (30%), and hyperreflexia items (30%).

In contrast to sham-treated individuals, patients in the real tDCS arm performed better on the PATA repetition task at each of their follow-up visits compared to baseline. Between-group differences were close to significance at T2 (95% CI of difference between groups: − 0.02 to 8.82, *p* = 0.051) and T4 (95% CI: − 0.34 to 6.71, *p* = 0.074) (Fig. 2D). No short-term or long-term treatment effects were observed for gait speed, 9HPT performance, and IPAQ scores.

### Static Posturography

When considering average values of three consecutive trials, absolute changes in amplitude and velocity of sway in the AP and ML directions did not significantly differ between both groups at T1 (Table 3). Likewise, none of the examined parameters in the best of three trials was modified by real tDCS. Somewhat surprisingly, the only significant change from baseline was observed in the sham group, as average anteroposterior CoP velocity in the best trial decreased in sham-treated individuals (*p* = 0.035).

**Table 3:** Static posturography performance at baseline and after two weeks (T1) in the real and sham tDCS group. The results are expressed in cm. One patient in the sham condition was unable to maintain normal quiet stance for more than 30 s and could therefore not partake

	** Real tDCS**		** Sham tDCS**	
	**Baseline**	** T1**	**Baseline**	** T1**
**Mean of 3 trials**				
AP sway RMS	0.67 ± 0.18	0.64 ± 0.20	0.66 ± 0.26	0.62 ± 0.20
ML sway RMS	0.89 ± 0.25	0.83 ± 0.24	0.90 ± 0.21	0.82 ± 0.17
AP sway range	4.04 ± 1.24	3.65 ± 1.33	4.16 ± 1.70	3.75 ± 1.42
ML sway range	5.03 ± 1.31	4.68 ± 1.28	5.49 ± 1.53	4.72 ± 1.14
AP speed RMS	3.30 ± 1.14	3.18 ± 1.21	4.02 ± 2.54	3.33 ± 1.57
ML speed RMS	3.65 ± 0.95	3.58 ± 1.23	4.03 ± 1.37	3.76 ± 1.11
Total path length	125.8 ± 33.5	122.8 ± 42.5	145.9 ± 68.6	128.5 ± 44.0
**Best of 3 trials**				
AP sway RMS	0.59 ± 0.14	0.56 ± 0.19	0.58 ± 0.19	0.53 ± 0.17
ML sway RMS	0.75 ± 0.22	0.74 ± 0.23	0.77 ± 0.16	0.73 ± 0.15
AP sway range	3.33 ± 0.92	3.08 ± 1.05	3.32 ± 1.34	3.01 ± 1.11
ML sway range	3.96 ± 1.01	3.99 ± 1.11	4.33 ± 1.25	4.04 ± 1.00
AP speed RMS	2.72 ± 0.89	2.81 ± 1.04	3.36 ± 1.82	2.83 ± 1.43^*^
ML speed RMS	3.16 ± 0.92	3.15 ± 1.12	3.42 ± 1.23	3.32 ± 1.03
Total path length	107.0 ± 31.0	111.0 ± 38.0	124.1 ± 50.5	113.7 ± 41.5

*AP* anteroposterior; *ML* mediolateral; *RMS* root mean square

^*^Significant change from baseline (*p* < 0.05)

### Patient-Reported and Non-Motor Outcomes

**Fig. 3:**
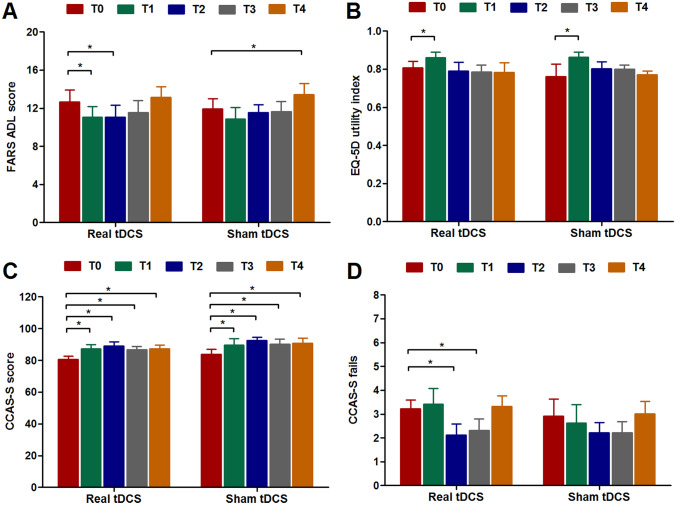
Patient-reported and cognitive outcomes at different time points. Panels (**A**) and (**B**) show group mean scores for the Activities of Daily Living part of the Friedreich Ataxia Rating Scale and the EQ-5D utility index, respectively, at baseline (T0) and after two weeks (T1), three months (T2), six months (T3), and twelve months (T4). Panels (**C**) and (**D**) show total score and number of failed tests at the cerebellar cognitive affective syndrome scale (CCAS-S) throughout the study. No treatment effects of real tDCS were observed for any of these outcome measures at any of the time points. ^*^Significant change from baseline (*p* < 0.05)

Directly after the last stimulation session, 50% of participants who had undergone real tDCS reported a slight overall improvement, as measured by the Patient Global Impression of Change scale, compared to 30% of participants who had undergone sham tDCS (*p* = 0.65). Back in their routine daily life, 50% of patients in the intervention group noticed relevant differences in physical functioning versus 20% of patients in the sham group (*p* = 0.35). Self-reported changes and duration of effects, as well as physician-rated changes in SARA score, are summarized for these individuals in Supplementary Table 4. ADL scores improved to a larger extent in the intervention group at T1, T2, and T3 than in the sham group but differences between both treatment arms did not reach significance (*p* > 0.15) (Fig. 3A).

SCA3 patients in both arms displayed a similar pattern of total CCAS-S score, that is, higher values at T1, T2, T3, and T4 compared to baseline (all *p* values < 0.01) without a treatment effect of real tDCS (Fig. 3C). Despite the application of parallel versions, our results probably indicate a general learning effect of repeated administration of the scale. Furthermore, there were no treatment effects at any of the time points for EQ-5D index (Fig. 3B), EQ-5D VAS, PHQ-9, iMCQ, and any of the POMS domain scores.

### Cerebellar Brain Inhibition

Due to technical issues, two patients were excluded from the CBI analysis. Resting motor threshold did not significantly increase after cerebellar anodal tDCS (*p* = 0.31). Similarly, mean change in MEP size ratio at 5 ms ISI did not differ between both treatment arms, indicating that repeated sessions of cerebellar anodal tDCS, on average, do not influence CBI in SCA3 patients (Table 4). MEP size ratio at 10 ms ISI decreased in the real stimulation group and increased in the sham group, yielding a statistically significant change in delta score (*p* = 0.004).

**Table 4:** Paired-pulse cerebellar-M1 TMS data at baseline and after two weeks (T1) in the real and sham tDCS group. Shown are group mean ratios between conditioned motor-evoked potential (MEP) amplitude in paired CS-TS trials and unconditioned MEP amplitude in TS-only trials

	**Sham tDCS**		**Real tDCS**	
	**Baseline**	**T1**	**Baseline**	**T1**
3 ms ISI	1.03 ± 0.14	1.06 ± 0.29	1.04 ± 0.29	1.03 ± 0.22
5 ms ISI	0.87 ± 0.17	0.98 ± 0.19	0.94 ± 0.07	0.96 ± 0.20
10 ms ISI	0.82 ± 0.15	1.01 ± 0.09	1.04 ± 0.27	0.90 ± 0.14

*ISI* interstimulus interval

### Blinding Control

Thoughts about treatment allocation at T1 in patients who had received real tDCS were distributed as follows: real stimulation (*n* = 3), sham stimulation (*n* = 2), unable to say (*n* = 5). Impression of group assignment in those who had received sham tDCS was distributed as follows: real stimulation (*n* = 1), sham stimulation (*n* = 4), unable to say (*n* = 5). In summary, 35% of patients were correct about their randomization status with an equal distribution among treatment arms (*p* = 0.44), indicating successful blinding.

## Discussion

In this randomized, double-blind, sham-controlled trial, we examined whether modulation of cerebellar excitability through anodal tDCS could benefit mildly to moderately affected SCA3 patients. In contrast to our hypothesis, a two-week regimen of daily cerebellar tDCS sessions did not significantly reduce overall ataxia severity compared to sham stimulation. No treatment effects were found for gait speed, manual dexterity, static posturography parameters, cognitive performance, various patient-reported outcomes, and CBI. Albeit exploratory, there was some indication for a difference between both groups in SARA speech and the PATA repetition task at six and twelve months of follow-up. While these findings need confirmation in future investigations, they might signify a long-term modulatory effect on articulation impairments, possibly as a result of tDCS-induced changes in medial lobule VI [49]. Furthermore, we noticed a treatment effect on INAS count after three and six months, which mainly involved the disappearance of mild dystonia, hyperreflexia, and urinary dysfunction in patients who received real tDCS.

Although we applied a similar study protocol with respect to electrode positions, electrode size, current intensity, and session duration, our results are at odds with those reported by Benussi et al. in a mixed cohort of twenty individuals with various SCA types, multiple system atrophy (MSA), and ataxia of other etiologies [21]. Overall, these investigators described a mean decrease in SARA score of 2.8 points after two weeks of cerebellar tDCS, which persisted for three months. As the largest reduction in ataxia severity was observed in individuals with less severe ataxia, we specifically included mildly to moderately affected patients in this trial. A comparison of baseline demographic and clinical variables between both studies indeed reveals that our SCA3 patients had a shorter disease duration (*p* = 0.008), lower SARA score (*p* = 0.006), higher gait speed (*p* = 0.011), and better 9HPT performance (dominant hand *p* = 0.003; non-dominant hand *p* < 0.001) than the SCA patients examined by Benussi and colleagues (Table 5) [21]. As the volume of cerebellar cortex that can be stimulated probably plays a key role in individuals with ataxia, it seems paradoxical that our patients, who had significantly lower clinical disease severity, did not experience beneficial effects at the group level, while the more severely affected SCA patients in the Benussi trial did.

**Table 5:** Comparisons of demographic and clinical characteristics between SCA3 patients in the present study and SCA patients in a previous trial by Benussi et al. [21]

	**Present study**	**Benussi et al. 2017**	**P value**
Age (y)	51.9 ± 10.0	45.3 ± 14.6	0.18
Disease duration (y)	8.0 ± 5.4	16.0 ± 9.4	0.008
SARA score	11.9 ± 3.9	17.9 ± 6.5	0.006
8 m walk test (s)	6.3 ± 2.1	10.8 ± 6.7	0.011
9-hole peg test, dominant hand (s)	31.6 ± 8.3	50.9 ± 23.1	0.003
9-hole peg test, non-dominant hand (s)	33.6 ± 6.4	55.0 ± 21.9	< 0.001

*SARA* Scale for the Assessment and Rating of Ataxia

Besides disparities in the extent of motor impairment, both disease-related and intervention-related factors could account for the overall lack of improvement. Regarding the former, one might hypothesize that degeneration of the dentate nuclei, brainstem structures, dorsal columns, and peripheral sensory nerves, all of which contribute to ataxia severity in SCA3 patients, cannot be reversed by modulation of cerebellar cortical excitability using tDCS [9, 11, 50]. As to tDCS-associated factors, it has been established that identical electrical doses delivered for a similar period of time through the same electrode montage may elicit heterogeneous physiological and behavioral responses [18, 51, 52]. Acknowledged sources of interindividual variability include genetic polymorphisms, age, attention, baseline level of function (and related ceiling or floor effects), physiological state of the targeted neuronal population, and differences in cortical geometry [51, 52]. Results of cerebellar tDCS are probably even more difficult to predict than stimulation over other brain regions because of the highly convoluted surface and complex cytoarchitecture of the cerebellar cortex [53, 54]. Still, modeling studies in healthy adults have demonstrated the highest electric field strengths in the posterior lobe, while the anteriorly located motor areas appear more difficult to reach [55, 56]. These anatomical considerations could be another reason for the overall absence of effects on ataxia severity in SCA3 patients.

Our data showed unexpected improvements in various outcome measures in the sham group. Intriguingly, the largest reduction in SARA score at T1 (i.e., 5 points) was found in a sham-treated patient, hinting at a considerable placebo effect or reflecting marked daily fluctuations in ataxia severity [57, 58]. Regular home assessments within a time span of two weeks including five of the eight SARA items recently revealed intraindividual differences up to 5.5 points, which is in line with our observation and seriously questions the utility of a single SARA score before and after any intervention in a clinical trial, especially if the sample size is relatively small [58]. Albeit not significant, it is interesting to note that half of the patients in the real tDCS group reported improvements in physical functioning back in their usual daily life versus 20% of patients in the sham group, while SARA score may suggest otherwise. Although this difference in self-perceived improvement should not be interpreted as a treatment effect, it does point to discrepancies between SARA scores and patient-reported outcomes [5, 59, 60].

Despite the lack of an overall treatment effect on ataxia severity, some of the patients in the real tDCS group showed a sustained decrease in SARA score lasting six or even twelve months, indicating interindividual variability in treatment response. In order to increase therapeutic efficacy, future studies are needed to identify predictors of long-standing symptomatic improvement.

In contrast to previous findings in a cohort with mixed ataxia etiologies, multiple sessions of tDCS in our study did not modulate cerebellar-M1 connectivity in SCA3 patients, as measured by paired-pulse TMS [21]. Reduced CBI levels have been linked to ataxia severity in SCA3 and — in parallel with our clinical findings — could thus not simply be restored by cerebellar anodal stimulation [41]. Although decreased CBI may arise from pathology anywhere along the cerebellothalamocortical tract, post-mortem investigations in SCA3 patients lead to the hypothesis that degeneration of the dentate nuclei plays a key role, which may not be overcome by modulation of the more superficially located cerebellar cortex [38, 39, 50]. The significant change observed at an interstimulus interval of 10 ms, which reflects the combination of a decreased MEP size ratio in the intervention group and an increased MEP size ratio in the sham group, is an unexpected finding, possibly indicative of larger intra-individual variability at an extended interval.

As of yet, conflicting results of single-session cerebellar tDCS have been described in individuals with ataxia [25, 61–65]. Notably, all investigations involved either single cases [61, 62] or etiologically heterogeneous groups of patients [25, 63–65], which precludes robust conclusions for specific entities. We therefore also examined if one session may induce a transient improvement in motor outcomes, but, again, found no supporting evidence in SCA3 (Supplementary Tables 5 and 6).

This study has several limitations. First, although the number of patients included would yield a theoretical power of more than 0.90 when considering previous results, a sample size of twenty participants is still relatively small. Second, SCA3 is not a pure cerebellar ataxia and effects of tDCS may have been masked by non-ataxia signs, such as polyneuropathy. Third, we used a clinical marker to estimate cerebellar reserve, namely SARA score. Validated, objective measures of functional cerebellar motor reserve that differentiate patients who are in the “non-restorable” stage from those in the “restorable” stage are urgently required [66, 67].

In conclusion, cerebellar anodal tDCS did not significantly improve various motor and non-motor outcome measures in mildly to moderately affected SCA3 patients compared to sham stimulation. Irrespective of the precise explanation, our findings call for further studies in other etiologically homogeneous groups of ataxia patients. These trials should include a sufficient follow-up duration to also ascertain possible long-term modulatory effects of tDCS on speech dysfunction. Finally, we cannot exclude the possibility that SCA3 patients may benefit from alternative cerebellar tDCS protocols, e.g., the application of higher current intensities, longer stimulation sessions, different electrode positions, or combined interventions with physical therapy [68, 69]. Likewise, following recent studies in individuals with essential tremor, one might hypothesize that cerebellar transcranial alternating current stimulation could be a more successful method to modulate activity along the cerebellothalamocortical tract and attain symptomatic relief in ataxia patients than tDCS [70, 71].

## Supplementary Information

Below is the link to the electronic supplementary material.

Supplementary file1 (PDF 216 KB)

Supplementary file2 (PDF 207 KB)

Supplementary file3 (PDF 508 KB)

Supplementary file4 (PDF 516 KB)

Supplementary file5 (PDF 46223 KB)

Supplementary file6 (PDF 509 KB)

Supplementary file7 (DOCX 55 KB)
