# Supplementary material for: Cerebellar Transcranial Direct Current Stimulation in Spinocerebellar Ataxia Type 3: a Randomized, Double-Blind, Sham-Controlled Trial
Source: Neurotherapeutics. 2022 May 2;19(4):1259–72. doi: 10.1007/s13311-022-01231-w (PMC9059914; doi:10.1007/s13311-022-01231-w)
Supplement: Supplementary file 7 — Supplementary file7 (DOCX 55 KB) [file 13311_2022_1231_MOESM7_ESM.docx]

**Supplementary data**

**A – Supplementary methods**

Detailed description of the different statistical models.

**B – Supplementary results**

Effects of a single tDCS session on motor outcomes in SCA3 patients.

**C – Supplementary tables**

Supplementary Table 1. Effects of real and sham cerebellar tDCS on motor and neurophysiological outcomes in SCA3 patients.

Supplementary Table 2. Short-term and long-term effects of real and sham cerebellar tDCS on cognitive and patient-reported outcome measures in SCA3 patients.

Supplementary Table 3. Differences in individual and aggregated SARA item scores between baseline and T1 in the real and sham tDCS group.

Supplementary Table 4. Self-reported changes of the five participants in the real tDCS group who noticed improvements in their daily activities after the two-week treatment regimen, along with their SARA scores at baseline and after two weeks, three months, six months, and twelve months.

Supplementary Table 5. Effects of a single session of real and sham cerebellar anodal tDCS on motor outcome measures in SCA3 patients.

Supplementary Table 6. Comparisons of demographic and clinical characteristics between SCA3 patients in the present study and SCA patients in a previous single-session report by Benussi and colleagues (2015).

**D – Supplementary figures**

Supplementary Figure 1. Flowchart of study participants.

**A – Supplementary methods**

In general, marginal models were found to provide a better fit than subject-specific models with random intercepts and slopes. For every outcome measure, we have evaluated the following three marginal models and selected the one with the best fit to the data:

1. Change from baseline with no adjustment for other variables.
2. Change from baseline, adjusting for the baseline value of an outcome measure.
3. Outcomes at each visit, including baseline.

In statistical terms, these three models took the following forms:

1. $\Delta=\alpha_{1}I+\alpha_{2}T_{2}+\alpha_{3}T_{3}+\alpha_{4}T_{4}+\beta_{1}T_{1}trt+\beta_{2}T_{2}trt+\beta_{3}T_{3}trt+\beta_{4}T_{4}trt$

where $\Delta$ represents the change from baseline of a particular outcome measure (e.g., SARA score) at time point $T_{i}$ in the sham group ($trt=0$) or intervention group ($trt=1$). The intercept is given by $I$, while $\alpha_{i}$ and $\beta_{i}$ represent the estimated regression coefficients of the model. For the majority of endpoints, patients had four Δ scores (i.e., Δ_1_, Δ_2_, Δ_3_, and Δ_4_), which reflect the respective changes from baseline (= T0) to T1 (2 weeks), T2 (3 months), T3 (6 months), and T4 (12 months). Mean changes in a particular outcome measure at a given time point in the sham arm and intervention arm can thus be conceptualized as follows:

| **Change from baseline at time point** $\boldsymbol{T}_{\boldsymbol{i}}$ | **Sham arm** | **Intervention arm** |
| --- | --- | --- |
| $\Delta_{1}$ | $\alpha_{1}$ | $\alpha_{1}\boldsymbol{+}\boldsymbol{\beta}_{\boldsymbol{1}}$ |
| $\Delta_{i} (i=2,3,4)$ | $\alpha_{1}+\alpha_{i}$ | $\alpha_{1}+\alpha_{i}+\boldsymbol{\beta}_{\boldsymbol{i}}$ |

This table clarifies that the $\beta_{i}$ coefficients indicate differences in change from baseline between the intervention and sham group at $T_{i}$. Since randomization has taken place, no structural between-group differences were present at baseline and, consequently, differences in change from baseline to $T_{i}$ represent the treatment effects of cerebellar tDCS at $T_{i}$. As these $\beta_{i}$ coefficients are of primary interest in the paper, we have reported their values, along with 95% confidence intervals (CIs), for each outcome measure in Table 2. Note that the importance of these model estimates lies in obtaining 95% CIs and *p* values that take into account the repeated measurements nature of the data, thereby achieving more precision and greater statistical power than simple t-test comparisons at each of the individual time points. In addition to $\beta_{i}$ coefficients and 95% CIs, we have included the observed mean group scores at each time point in both treatment arms in Figures 1 to 3 and Supplementary Tables 1 and 2.

1. $\Delta=\alpha_{1}I+\alpha_{2}T_{2}+\alpha_{3}T_{3}+\alpha_{4}T_{4}+\beta_{1}T_{1}trt+\beta_{2}T_{2}trt+\beta_{3}T_{3}trt+\beta_{4}T_{4}trt$ + $\gamma$*[base covar]*

where *[base covar]* reflects the value of the covariate at baseline and $\gamma$ represents a third regression coefficient that captures the impact of the covariate on the change score.

| **Change from baseline at time point** $\boldsymbol{T}_{\boldsymbol{i}}$ | **Sham arm** | **Intervention arm** |
| --- | --- | --- |
| $\Delta_{1}$ | $\alpha_{1}+ \gamma\cdot[base covar]$ | $\alpha_{1}\boldsymbol{+}\boldsymbol{\beta}_{\boldsymbol{1}}$ $+ \gamma\cdot[base covar]$ |
| $\Delta_{i} (i=2,3,4)$ | $\alpha_{1}+\alpha_{i}$ + $\gamma\cdot[base covar]$ | $\alpha_{1}+\alpha_{i}+\boldsymbol{\beta}_{\boldsymbol{i}}$ $+ \gamma\cdot[base covar]$ |

This model allows the mean change to depend on the baseline value of the covariate in the subject. In particular, it estimates the treatment effect $\beta_{i}$ at time point $T_{i}$ with a correction for the baseline value (i.e., the difference between patients in the intervention group and sham group at $T_{i}$ if they had the same baseline value of the covariate).

1. $Y=\alpha_{0}I+\alpha_{1}T_{1}+\alpha_{2}T_{2}+\alpha_{3}T_{3}+{\alpha_{4}T_{4}+\beta}_{1}T_{1}trt+\beta_{2}T_{2}trt+\beta_{3}T_{3}trt+\beta_{4}T_{4}trt$

where Y represents the mean value of an outcome measure at T0, T1, T2, T3, and T4.

| **Time point** | **Sham arm** | **Intervention arm** |
| --- | --- | --- |
| $T_{0}$ | $\alpha_{0}$ | $\alpha_{0}$ |
| $T_{i} (i=1,2,3,4)$ | $\alpha_{0}+ \alpha_{i}$ | $\alpha_{0}+ \alpha_{i}+ \boldsymbol{\beta}_{\boldsymbol{i}}$ |

Note that due to randomization there is no structural difference between groups at baseline: the mean is estimated by $\alpha_{0}$ in both arms. Otherwise stated, any difference observed at baseline is due to chance and the true mean is best estimated by taking the mean of all patients at baseline.

**B – Supplementary results**

**Effects of a single tDCS session on motor outcomes in SCA3 patients**

Absolute change in SARA score did not significantly differ between SCA3 patients treated with real tDCS (-0.65 ± 1.08; 95% confidence interval [CI] -1.42 to 0.12) and those who received sham stimulation (0.00 ± 1.63; 95% CI -1.17 to 1.17) (*t*(18) = 1.05, *p* = 0.31, Cohen’s *d* = 0.47). Clinically meaningful improvement was found in 30% of participants in both groups. The absolute mean decrease of 0.65 points in the real tDCS arm occurred as a result of better scores at stance (-0.40) and fast alternating hand movements items (-0.25).

In line with the aforementioned findings, real tDCS did not significantly enhance gait speed (*p* = 0.74), 9HPT performance (dominant hand: *p* = 0.99; non-dominant hand: *p* = 0.60), or articulation speed (*p* = 0.32) as compared with sham stimulation. Lastly, there were no differences between both treatment groups in amplitude and velocity of sway in the anteroposterior and mediolateral directions or in total CoP path length, neither in the best trial nor in all three trials combined (all *p* > 0.15).

**C – Supplementary tables**

|  | **T0**  **Baseline** | **T1**  **2 weeks** | **T2**  **3 months** | **T3**  **6 months** | **T4**  **12 months** |
| --- | --- | --- | --- | --- | --- |
| **Real tDCS** |  |  |  |  |  |
| *Ataxia severity* |  |  |  |  |  |
| SARA score | 11.3 ± 3.2 | 10.5 ± 3.7 | 10.3 ± 3.5 | 10.4 ± 3.6 | 10.5 ± 3.2 |
| SARA score, percent change | - | -8.9 ± 9.1^*^ | -7.5 ± 24.3 | -6.6 ± 25.7 | -4.2 ± 25.2 |
| SARA axial | 4.9 ± 1.4 | 4.4 ± 1.6 | 4.3 ± 1.8 | 4.7 ± 1.6 | 4.3 ± 1.7 |
| SARA appendicular | 4.2 ± 1.7 | 4.1 ± 1.3 | 3.9 ± 1.5 | 4.0 ± 1.5 | 4.3 ± 1.3 |
| SARA speech | 2.2 ± 0.6 | 2.0 ± 0.8 | 2.1 ± 1.0 | 1.7 ± 0.8^*^ | 1.9 ± 0.6 |
| *Quantitative motor tests and extracerebellar signs* |  |  |  |  |  |
| 8MWT (s) | 5.7 ± 0.9 | 5.6 ± 0.9 | 5.6 ± 0.8 | 5.9 ± 0.9 | 6.1 ± 1.1^a^ |
| 9HPT, dominant hand (s) | 32.1 ± 7.4 | 30.5 ± 6.1 | 29.7 ± 5.9 | 30.7 ± 5.6 | 32.3 ± 7.9^b^ |
| 9HPT, nondominant hand (s) | 33.3 ± 5.0 | 32.7 ± 6.2 | 32.2 ± 5.6 | 32.0 ± 5.3 | 33.6 ± 5.6^b^ |
| PATA rate | 27.2 ± 5.9 | 30.3 ± 6.5^*^ | 31.4 ± 7.4^*^ | 29.8 ± 6.3 | 29.1 ± 5.1^*b^ |
| INAS count | 5.9 ± 1.7 | 5.3 ± 1.9^*^ | 5.0 ± 1.3 | 4.8 ± 1.9^*^ | 6.3 ± 1.2^b^ |
| *Neurophysiological outcome measures* |  |  |  |  |  |
| TMS – rMT (%) | 40.2 ± 3.6 | 40.9 ± 3.5 | - | - | - |
| TMS – CBI | 0.94 ± 0.07 | 0.96 ± 0.20 | - | - | - |
|  |  |  |  |  |  |
| **Sham tDCS** |  |  |  |  |  |
| *Ataxia severity* |  |  |  |  |  |
| SARA score | 12.5 ± 4.7 | 11.5 ± 4.4^*^ | 11.4 ± 5.0 | 12.1 ± 5.1 | 12.3 ± 4.9 |
| SARA score, percent change | - | -9.0 ± 14.7^*^ | -12.1 ± 16.8 | -5.9 ± 14.1 | -2.7 ± 8.4 |
| SARA axial | 5.6 ± 2.4 | 5.2 ± 2.0 | 5.2 ± 2.4 | 5.6 ± 2.4 | 5.4 ± 2.5 |
| SARA appendicular | 5.2 ± 2.2 | 4.6 ± 2.0^*^ | 4.4 ± 2.1 | 4.7 ± 2.1 | 5.1 ± 2.1 |
| SARA speech | 1.7 ± 0.7 | 1.7 ± 0.9 | 1.8 ± 1.0 | 1.8 ± 1.0 | 1.8 ± 1.0 |
| *Quantitative motor tests and extracerebellar signs* |  |  |  |  |  |
| 8MWT (s) | 6.8 ± 2.8 | 6.3 ± 2.2 | 6.6 ± 2.3 | 7.0 ± 3.2 | 8.1 ± 4.2^*b^ |
| 9HPT, dominant hand (s) | 31.1 ± 9.5 | 28.4 ± 7.2^*^ | 29.5 ± 7.6 | 30.4 ± 9.7 | 31.7 ± 8.3^b^ |
| 9HPT, nondominant hand (s) | 33.9 ± 7.8 | 33.1 ± 10.1 | 33.0 ± 8.0 | 34.5 ± 9.8 | 35.1 ± 8.4^b^ |
| PATA rate | 28.1 ± 6.1 | 29.8 ± 6.9 | 27.9 ± 5.1 | 28.6 ± 6.5 | 26.9 ± 5.7^b^ |
| INAS count | 5.8 ± 1.5 | 5.9 ± 1.4 | 6.6 ± 1.1 | 7.3 ± 1.3^*^ | 7.4 ± 2.3^*b^ |
| *Neurophysiological outcome measures* |  |  |  |  |  |
| TMS – rMT (%) | 40.6 ± 4.0 | 40.5 ± 4.1 | - | - | - |
| TMS – CBI | 0.87 ± 0.17 | 0.98 ± 0.19 | - | - | - |

**Supplementary Table 1.** Effects of real and sham cerebellar tDCS on motor and neurophysiological outcomes in SCA3 patients.

^*^ Significant change from baseline (*p* < 0.05).

^a^ Data from 7 patients (two visits in March/April 2020 had to be cancelled because of the COVID-19 pandemic; another participant had sustained knee injury from a fall the week before her last follow-up visit, which precluded her from performing the 8MWT).

^b^ Data from 8 patients (two visits in March/April 2020 had to be cancelled because of the COVID-19 pandemic).

SARA = Scale for the Assessment and Rating of Ataxia; 8MWT = 8 m walk test; 9HPT = nine-hole peg test; INAS = Inventory of Non-Ataxia Signs; rMT = resting motor threshold; CBI = cerebellar brain inhibition.

|  | **T0**  **Baseline** | **T1**  **2 weeks** | **T2**  **3 months** | **T3**  **6 months** | **T4**  **12 months** |
| --- | --- | --- | --- | --- | --- |
| **Real tDCS** |  |  |  |  |  |
| *Neuropsychological outcome measures* |  |  |  |  |  |
| CCAS-S, total score | 80.3 ± 7.0 | 86.9 ± 9.7^*^ | 88.7 ± 9.4^*^ | 86.4 ± 7.5^*^ | 87.0 ± 7.9^*^ |
| CCAS-S, failed tests | 3.2 ± 1.2 | 3.4 ± 2.1 | 2.1 ± 1.5^*^ | 2.3 ± 1.6^*^ | 3.3 ± 1.5 |
| *Patient-reported outcome measures* |  |  |  |  |  |
| EQ-5D VAS score | 77.8 ± 12.4 | 76.0 ± 11.6 | 68.5 ± 8.9^*^ | 66.7 ± 17.7^*^ | 70.3 ± 14.2^*^ |
| EQ-5D utility index | 0.80 ± 0.11 | 0.86 ± 0.10^*^ | 0.79 ± 0.15 | 0.78 ± 0.13 | 0.78 ± 0.17 |
| PHQ-9 score | 3.8 ± 3.4 | 2.9 ± 4.7 | 4.3 ± 3.9 | 4.7 ± 4.5 | 4.0 ± 4.3 |
| POMS fatigue | 4.5 ± 3.9 | 3.9 ± 3.7 | 5.7 ± 3.4 | 5.0 ± 4.5 | 3.9 ± 3.6 |
| POMS depression | 3.3 ± 2.7 | 2.1 ± 3.0^*^ | 3.2 ± 3.3 | 2.4 ± 3.2 | 3.0 ± 2.9 |
| POMS anger | 3.6 ± 4.2 | 2.1 ± 2.8 | 4.2 ± 4.4 | 3.4 ± 5.2 | 3.1 ± 3.2 |
| POMS tension | 1.8 ± 2.0 | 0.8 ± 1.3 | 2.5 ± 2.7 | 1.4 ± 1.8 | 2.1 ± 2.3 |
| POMS vigor | 12.4 ± 4.4 | 13.1 ± 4.4 | 11.4 ± 4.1 | 11.6 ± 3.0 | 11.5 ± 3.2 |
| FARS ADL score | 12.6 ± 4.1 | 11.0 ± 3.7^*^ | 11.0 ± 4.2^*^ | 11.5 ± 4.1 | 13.1 ± 3.7 |
| iMCQ score (euro) | 636 ± 409 | - | - | - | 823 ± 532 |
| IPAQ (MET minutes per week) | 2742 ± 2512 | - | 1908 ± 2119^*^ | - | 1560 ± 1196 |
|  |  |  |  |  |  |
| **Sham tDCS** |  |  |  |  |  |
| *Neuropsychological outcome measures* |  |  |  |  |  |
| CCAS-S, total score | 83.4 ± 11.2 | 89.3 ± 13.6^*^ | 92.1 ± 7.5^*^ | 89.8 ± 11.2^*^ | 90.4 ± 10.8^*^ |
| CCAS-S, failed tests | 2.9 ± 2.3 | 2.6 ± 2.5 | 2.2 ± 1.4 | 2.2 ± 1.5 | 3.0 ± 1.7 |
| *Patient-reported outcome measures* |  |  |  |  |  |
| EQ-5D VAS score | 67.6 ± 19.0 | 71.4 ± 21.0 | 67.2 ± 17.6 | 68.0 ± 14.2 | 66.4 ± 14.3 |
| EQ-5D utility index | 0.76 ± 0.21 | 0.86 ± 0.09^*^ | 0.80 ± 0.12 | 0.80 ± 0.08 | 0.77 ± 0.07 |
| PHQ-9 score | 3.6 ± 2.5 | 3.5 ± 3.0 | 4.4 ± 3.5 | 3.9 ± 2.2 | 3.3 ± 1.6 |
| POMS fatigue | 5.2 ± 4.9 | 4.8 ± 7.1 | 5.6 ± 5.9 | 6.9 ± 5.5 | 4.7 ± 5.2 |
| POMS depression | 1.9 ± 2.6 | 0.9 ± 1.7^*^ | 1.7 ±2.1 | 1.1 ± 2.0 | 1.1 ± 1.5 |
| POMS anger | 1.6 ± 3.5 | 0.5 ± 1.1 | 1.7 ± 2.2 | 0.6 ± 1.0 | 0.6 ± 1.7 |
| POMS tension | 1.7 ± 1.3 | 1.0 ± 1.3 | 1.8 ± 2.5 | 1.4 ± 2.0 | 2.0 ± 2.0 |
| POMS vigor | 11.2 ± 4.0 | 11.9 ± 4.3 | 11.4 ± 4.8 | 12.1 ± 3.4 | 12.2 ± 4.2 |
| FARS ADL score | 11.9 ± 3.5 | 10.8 ± 4.0 | 11.5 ± 2.8 | 11.6 ± 3.5 | 13.4 ± 3.7^*^ |
| iMCQ score (euro) | 719 ± 513 | - | - | - | 861 ± 851 |
| IPAQ (MET minutes per week) | 2361 ± 1851 | - | 2126 ± 1513 | - | 1593 ± 1600 |

**Supplementary Table 2.** Short-term and long-term effects of real and sham cerebellar tDCS on cognitive and patient-reported outcome measures.

^*^ Significant change from baseline (*p* < 0.05).

CCAS-S = cerebellar cognitive affective syndrome scale; PHQ-9 = Patient Health Questionnaire-9; POMS = Profile of Mood States; FARS = Friedreich Ataxia Rating Scale; ADL = activities of daily living; iMCQ = Institute for Medical Technology Assessment (iMTA) Medical Consumption Questionnaire; IPAQ = International Physical Activity Questionnaire; MET = Metabolic Equivalent of Task.

|  | **Real tDCS** | **Sham tDCS** |
| --- | --- | --- |
| **Individual items** |  |  |
| Gait | 0.10 ± 0.32 | -0.10 ± 0.57 |
| Stance | -0.60 ± 0.97 | -0.10 ± 0.32 |
| Sitting | 0.00 ± 0.47 | -0.20 ± 0.42 |
| Speech | -0.20 ± 0.42 | 0.00 ± 0.47 |
| Finger chase | 0.05 ± 0.16 | -0.15 ± 0.24 |
| Nose-finger test | 0.10 ± 0.46 | 0.00 ± 0.33 |
| Fast alternating hand movements | -0.25 ± 0.68 | -0.30 ± 0.59 |
| Heel-shin slide | -0.05 ± 0.60 | -0.15 ± 0.63 |
| **Aggregated domains** |  |  |
| Axial | -0.50 ± 1.08 | -0.40 ± 1.08 |
| Upper limb | -0.10 ± 0.70 | -0.45 ± 0.60 |
| Appendicular | -0.15 ± 0.67 | -0.60 ± 0.84 |
| Total | -0.85 ± 0.94 | -1.00 ± 1.86 |

**Supplementary Table 3.** Differences in individual and aggregated SARA item scores between baseline and T1 in the real and sham tDCS group.

| **Age (y)** | **Sex** | **Reported changes at home** | **Baseline SARA score** | **Subsequent SARA scores** |
| --- | --- | --- | --- | --- |
| 47 | M | Improvement in walking for approximately 4 weeks | 15.5 | 14.5, 13, 12, and 11.5 |
| 65 | M | Considerable improvement in walking (he could walk 2.5 km at the golf course and did not require the buggy he normally used) and standing still for approximately 5 days; absence of muscle cramps for 10 days | 12 | 11, 8, 7.5, and 10 |
| 51 | F | Increased stability during walking and standing still and less dizziness for 2 months | 13 | 13, 13.5, 13.5, and 13.5 |
| 35 | F | Improvement in walking and less choking for approximately 1.5 weeks | 11 | 9.5, 9, 9, and 10 |
| 62 | M | Improvement in walking for 7 months | 9.5 | 7, 4.5, 4.5, and 5 |

**Supplementary Table 4.** Self-reported changes of the five participants in the real tDCS group who noticed improvements in their daily activities after the two-week treatment regimen, along with their Scale for the Assessment and Rating of Ataxia (SARA) scores at baseline and after two weeks, three months, six months, and twelve months.

|  | **Real tDCS** | | **Sham tDCS** | |  |
| --- | --- | --- | --- | --- | --- |
|  | **Before** | **After** | **Before** | **After** | **P value** |
| **SARA score** | 11.30 ± 3.18 | 10.65 ± 3.33 | 12.5 ± 4.67 | 12.5 ± 4.13 | 0.31 |
| **8MWT (s)** | 5.70 ± 0.91 | 5.57 ± 0.86 | 6.84 ± 2.82 | 6.54 ± 2.22 | 0.74 |
| **9HPT, dominant hand (s)** | 32.10 ± 7.42 | 32.49 ± 6.68 | 31.09 ± 9.54 | 31.48 ± 9.41 | 0.99 |
| **9HPT, nondominant hand (s)** | 33.30 ± 5.02 | 32.33 ± 4.78 | 33.88 ± 7.79 | 32.26 ± 8.14 | 0.60 |
| **PATA repetition rate** | 27.20 ± 5.87 | 29.35 ± 7.61 | 28.05 ± 6.08 | 28.25 ± 5.62 | 0.32 |

**Supplementary Table 5.** Effects of a single session of real and sham cerebellar anodal tDCS on motor outcome measures in SCA3 patients.

Data are presented as mean ± standard deviation.

SARA = Scale for the Assessment and Rating of Ataxia; 8MWT = 8 meter walk test; 9HPT = nine-hole peg test.

|  | Present study | Benussi et al. 2015 | T value | P value |
| --- | --- | --- | --- | --- |
| Age (y) | 51.9 ± 10.0 | 43.0 ± 13.0 | 1.95 | 0.06 |
| Age at onset (y) | 43.9 ± 9.2 | 30.3 ± 8.4 | 3.62 | 0.001 |
| Disease duration (y) | 8.0 ± 5.4 | 12.8 ± 10.5 | -1.61 | 0.12 |
| SARA score | 11.9 ± 3.9 | 18.4 ± 7.9 | -2.92 | 0.007 |
| 8 m walk test (s) | 6.27 ± 2.12 | 15.83 ± 5.97 | -6.37 | < 0.001 |
| 9-hole peg test (s)^#^ | 32.59 ± 7.40 | 61.76 ± 32.48 | -3.87 | 0.001 |

**Supplementary Table 6**. Comparisons of demographic and clinical characteristics between SCA3 patients in the present study and SCA patients in a previous single-session report by Benussi and colleagues (2015).

Data are presented as mean ± standard deviation.

^#^ average performance of both hands

**D – Supplementary figures**

Supplementary Figure 1. Flowchart of study participants.

**CONSORT Flow Diagram**

Analyzed

(n = 10)

Analyzed

(n = 10)

Discontinued intervention (n = 0)

Lost to follow-up (n = 0)

Allocated to real tDCS

(n = 10)

Discontinued intervention (n = 0)

Lost to follow-up (n = 0)

Allocated to sham tDCS

(n = 10)

Randomized

(n = 20)

Assessed for eligibility

(n = 20)

## Enrollment

## Analysis

## Allocation

## Follow-up
